
JOURNAL INFORMATION
==============================
NLM Title Abbreviation: ScientificWorldJournal
Journal ID: ScientificWorldJournal
NLM Journal ID: 101131163
Journal ID: TSWJ

The Scientific World Journal

ISSN: 2356-6140
EISSN: 1537-744X
Publisher: Wiley

ARTICLE INFORMATION
==============================
PMCID: PMC5936507
PMID: 16299643
DOI: 10.1100/tsw.2005.120
Article ID: 135820
Article version: 1
Subjects: Directions in Science

Remote Islands Reveal Rapid Rise of Southern Hemisphere Sea Debris

Barnes David K.A.
British Antarctic Survey, Natural Environment Research Council, High Cross, Cambridge, CB3 OET, U.K. and University College Cork, Cork, Ireland
Publication date: 2005
Electronic publication date: 2005 Nov 16
Volume: 5
First page: 915
Last page: 921
Received 2005 Nov 3; Accepted 2005 Nov 11
Copyright: Copyright © 2005 David K.A. Barnes.
Copyright year: 2005
License: This is an open access article distributed under the Creative Commons Attribution License, which permits unrestricted use, distribution, and reproduction in any medium, provided the original work is properly cited.
License URL: https://creativecommons.org/licenses/by/3.0/

==============================
Directions in Science
*TheScientificWorldJOURNAL* (2005) 5, 915–921
ISSN 1537-744X; DOI 10.1100/tsw.2005.120

### Remote Islands Reveal Rapid Rise of Southern Hemisphere Sea Debris

David K.A. Barnes

*British Antarctic Survey, Natural Environment Research Council, High Cross, Madingley Road, Cambridge, CB3 OET, U.K. and University College Cork, Cork, Ireland*

**E-mail:** dkab@bas.ac.uk

*Received November 3, 2005; Accepted November 11, 2005; Published November 16, 2005*

---

In the middle of the last century, plastics heralded a new era; by the 1980s, their legacy, marine litter, was reaching the most remote island shores[1] and increasing rapidly[2]. However, accumulation of plastics and other debris was still relatively inconsequential on shores in the Southern Ocean[3]. The debris data presented here demonstrate, for the first time, unequivocal spatial and temporal trends in accumulation across the entire southern hemisphere. There is a clear relationship with latitude from more than 2 items per meter per year at the equator to less than 0.01 item per meter per year on the most southern shores. Human habitation patterns can almost entirely ($r^2 > 97\%$) explain this cline. The data demonstrate both increased and accelerated artefact accumulation on remote shores over more than 3 decades. The rate of change is, however, most extreme at cold temperate/polar latitudes. Plastics are pushing polewards; the miracle material could transform Antarctica's shores from wilderness to wasteland.

Ability and cost of mass production of plastic items made them disposable and their versatility and durability made them indispensable. Two decades ago, more than 8 million items per day were estimated to be entering the sea, of which about 8% were plastics[4]. A recent paper in *Science*[5] showed the rapid increase in microplastics recorded in the water column from the 1960s and 1970s to the 1980s and 1990s around the U.K. The authors of the study also showed that these microplastics were accumulating on shores and particularly in subtidal environments throughout the U.K. coastline. The very attributes that made plastics so ubiquitous are now ensuring their marine persistence, but it is increasingly apparent that this is not limited to sea and shores in the industrial world. Other recent studies of macrodebris at sea[6,7] have shown that more than one item washes ashore per meter per year on remote islands across the Indian and Atlantic oceans. Not long ago, there was little southern hemisphere information on the invasion of the sea and coast by marine debris, but there is now public domain information on flotsam strandings from sites widespread across austral latitudes and longitudes (Fig. 1).

As well as the large-scale habitat alteration effects of marine pollution, seaborne items of litter also have important individual consequences. Debris, particularly persistent plastics, can poison, choke, or starve, but also act as a transport and dispersal system[8]. Flotsam has, therefore, the potential to carry non-native or invasive 'pest' species over long distances and a wide range of locations. The scale of organism transport is huge; in some areas, tens to hundreds of thousands of macro-organisms may be carried per square meter of debris[7]. In addition, sea debris can be transporting micro-organisms such as harmful algal blooms[9]. Attention and concern has typically centered around the more densely used northern hemisphere oceans and seas, but marine debris is booming in the southern hemisphere and reaches into the Southern Ocean (Fig. 2).

©2005 with author.

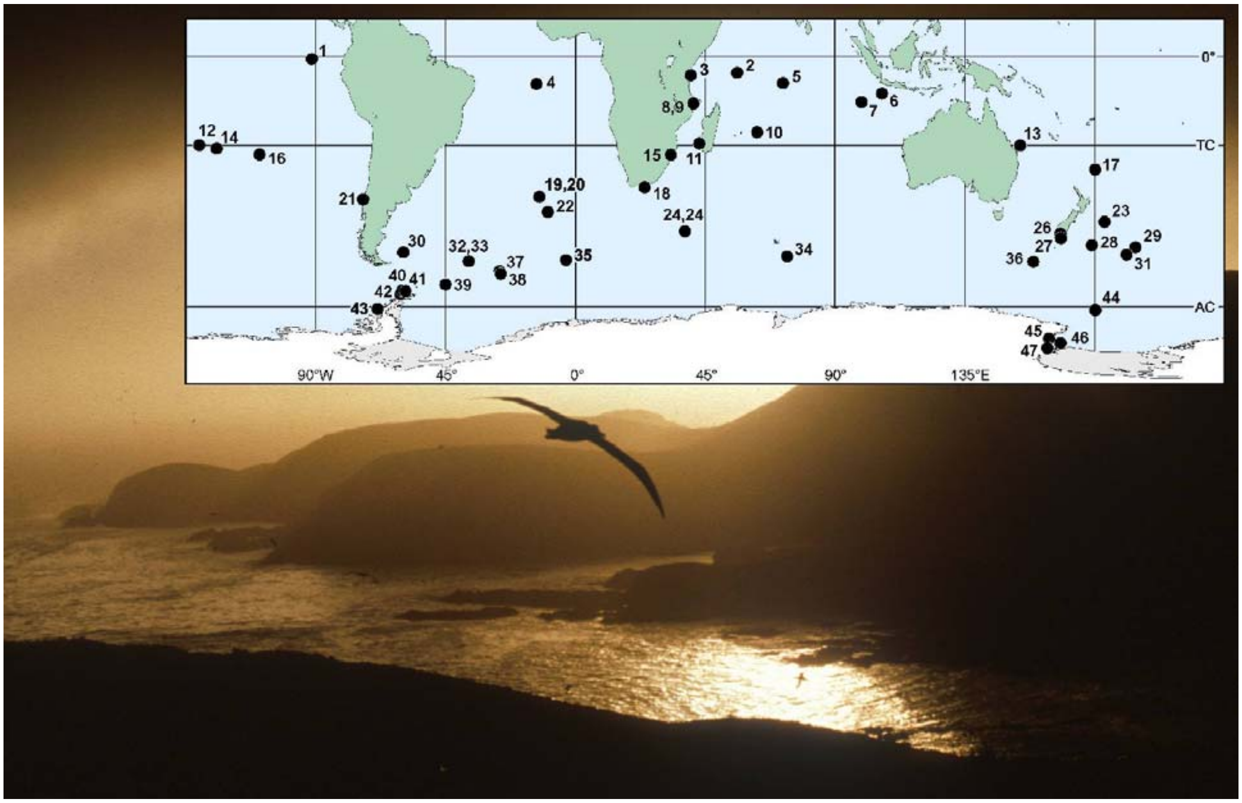

**FIGURE 1.** Accumulation of marine debris, plastics in particular, are still increasing on remote islands in the southern hemisphere that have been surveyed (numbered by increasing latitude - insert). Deposition of this oceanic flotsam on shores around the margins of the Southern Ocean, such as Bird Island (main picture), is accelerating and, in 2000, reached levels one to two orders of magnitude higher than in the previous decade. This poses an increasingly significant threat to seabirds such as wandering albatross (shown). Photo by K. Reid.

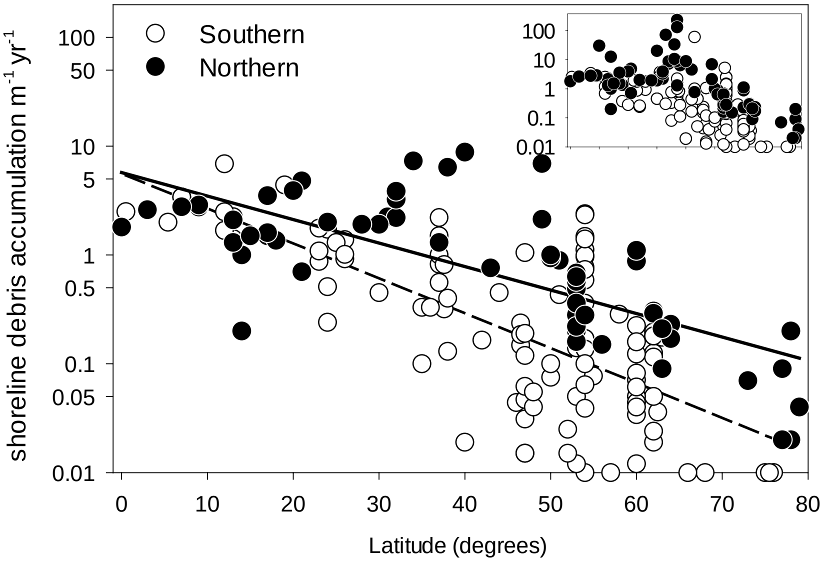

**FIGURE 2.** Debris accumulation on shores across the globe. Values are calculated from the literature[1,2,3,6,7,8,12,13,14,15,16,17,18,19]. Hemispheres are identified by insert legend. Relationships are significant (ANOVA, df = 1, F ratio = 26.5 and 37.4 for north and south, respectively, both $p < 0.001$, both $r^2 = 40\%$). Relationships north and south are significantly different (GLM ANOVA, df 1,1, F ratio 21.7, $p < 0.001$). Insert (right) includes data for urban sites.

Nonanthropogenic debris (driftwood and pumice) was reported to have crossed the Polar Frontal Zone (PFZ) in both directions[10,11] several decades ago. Debris on some southeastern Indian and west Pacific shores have even been reported to have a Southern Ocean source[12]. Clearly, even the PFZ (considered to be the most substantial surface oceanographic barrier) is not effective as an obstacle to flotsam. Aesthetics of stranding marine litter alone may have major commercial implications. Cleaning efforts on South African beaches have, on average, doubled each decade[13]. Despite some high profile reports of the scale and pattern of debris increase at specific locations[1,2], as well as the vast distances the debris may cover[6,7,8,9,10,11], scientific attention waned while litter waxed[2]. Following the recent resurgence of interest and concern about marine plastics and their consequences[5,7,8,9] and the increasing intensity of surveys in the southern hemisphere, aspects of panaustral accumulation patterns have been collated from the literature and some trends presented here.

The now-widespread marine litter surveys on continental shores have shown that debris density decreases rapidly away from urban centers[11]. No other relationship is obvious in time and space (e.g., latitude, Fig. 2 insert). Since the 1980s, there has, however, been sporadic research on the marine debris washing up on remote shores of islands in the Atlantic, Indian, Pacific, and Southern oceans[1,2,3,6,7,8,12,13,14,15,16,17,18,19,20]. Despite this, few geographic trends have previously been constructed, either by latitude or longitude (but see [7,8]). Meaningful transocean comparisons remain problematical due to the restricted range of data (and study islands) in certain oceans, such as the south Atlantic. Nevertheless, combination of both 10 years of sampling (from tropical Ascension at 7ºS to Adelaide Island, Antarctica at 68ºS) with the broad, but often isolated, literature shows that there is a clear trend in marine debris accumulation with latitude (Fig. 2, main graph). The highest levels, in the tropics to warm temperate regions, peak at about 2.5 items per meter per year. Variability in accumulation of debris on (mostly remote) southern hemisphere islands is at the scale of about three orders of magnitude. There are a myriad of potential factors, which might be expected to induce high variability, such as degree of isolation and compass direction of beaches. The best-fit relationship indicated (Fig. 2 main graph), however, explains virtually half of total data variability. Thus, it can be concluded that, overall, latitude (or a correlate of) is the biggest predictor of debris accumulation rates. Time, though, also proved to be an important source of variability.

There was mixed evidence in the 1970s on whether or not marine debris was increasing or decreasing[14,15,16] in southern seas. Reports in the 1980s suggested that not only was marine litter reaching even remote islands in most southern hemisphere oceans[3,14,16] (Fig. 3), but also that it was increasing at the few sites that were monitored[2,15,16]. By the 1990s, the Commission for Conservation of Antarctic Marine Living Resources (CCAMLR) had instigated programs for monitoring shore debris, although some signatories had already been doing so[16]. The data in Fig. 2 show a strong and relatively simple spatial relationship of marine debris accumulation. Multiyear studies at some remote island sites, such as Inaccessible Island (South Atlantic)[2], have found clear time trends as well. Spatial and temporal relationships were analyzed by calculating means of accumulation values pooled by each 5 degrees of latitude and by decade (Fig. 4). Differences between 1970s and 1980s accumulation levels were not apparent in the southern hemisphere (unlike in the north[5]), though little data are available from the former. Trend curves for the two following decades were clearly different. however, both from each other and earlier data. The remote island study localities had higher debris accumulation rates with increasing time, but the rate of increase was greatest in and around the Southern Ocean. The amount of debris washing ashore in the tropics and subtropics had typically doubled from the 1980s to 2000s. On south Atlantic island shores, rates had, however, increased by an order of magnitude and nearly two orders of magnitude by 60 degrees south.

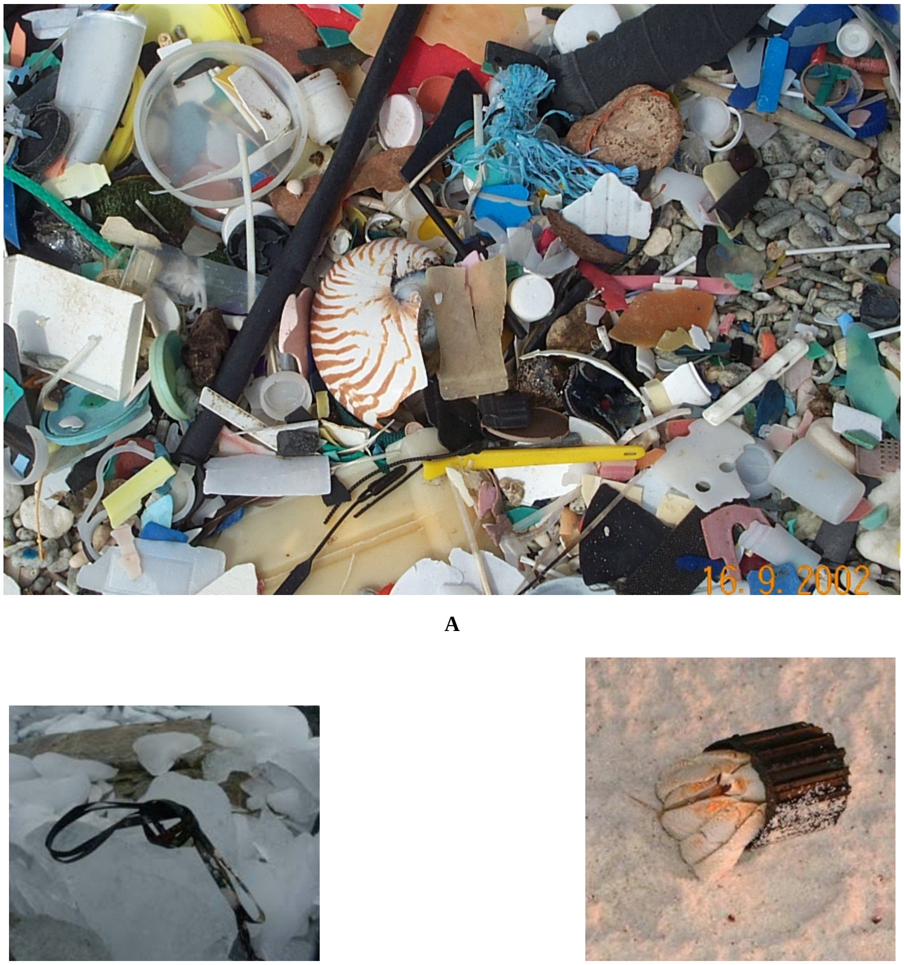

A

B C

**FIGURE 3**. Shore signs of the times. Debris, mostly plastic, washed ashore on remote Cocos Island, Indian Ocean (A). The most southerly colonized debris was found near Adelaide Island, Antarctica (B). At some places, like Diego Garcia, plastic tops are becoming a significant source of homes for hermit crabs (C). Photo 3a by Sven Sewell and Photo 3c by Thomas Lawson.

Most of the flotsam washed ashore is anthropogenic, though this has been shown to increase with increasing latitude[8]. Accumulation rates of this fraction (man-made) debris seem to be very closely correlated with human population levels for each 10 degrees of latitude from the equator to near pole (Fig. 5). Differential surface area from the tropics to pole does not appear to be a confounding factor as accumulation rates have a similarly high correlation with human population density (Fig. 5 insert). Such a close correlation must cast some doubt on the significance and extent of export of seaborne rubbish from the ‘dirty seas’ of the northern, more heavily populated hemisphere. Despite transoceanic travel of some items[1,2,10,11], it seems likely that culpability begins at home; the litter legacy is literally in our littoral. In contrast to the proportions estimated to be entering the oceans[4], plastic was generally the principal constituent of shore debris. Between 47 and 100% of artifacts were plastics, except at the Snares Islands (south of New Zealand)[15] and the high Antarctic islands in the Ross Sea[3]. This variability, as with debris accumulation rates, formed a highly significant cline with latitude (Fig. 6). In contrast to overall debris trends, however, the proportion of plastics increased with increasing latitude up to ~60ºS. This seems likely to be explained by the buoyancy and durability of plastics enabling them to potentially travel large distances. Despite the undoubted export of plastics from industrialized to remote regions a

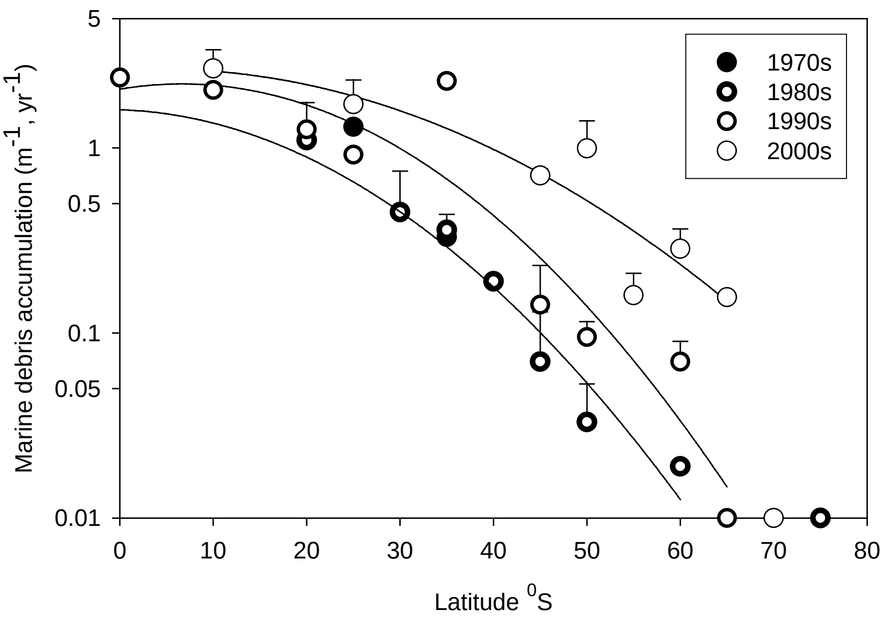

**FIGURE 4.** Mean macrodebris accumulation rates with latitude and time (decade). Data sources are as for Fig. 2. Means are shown with standard error for each 5 degrees of latitude. Fitted curves are all quadratic regressions, df 2, F ratio 28.4, 11.9, and 22.8; p = 0.01, 0.02, and 0.01 for the 1980, 1990, and 2000 decades, respectively.

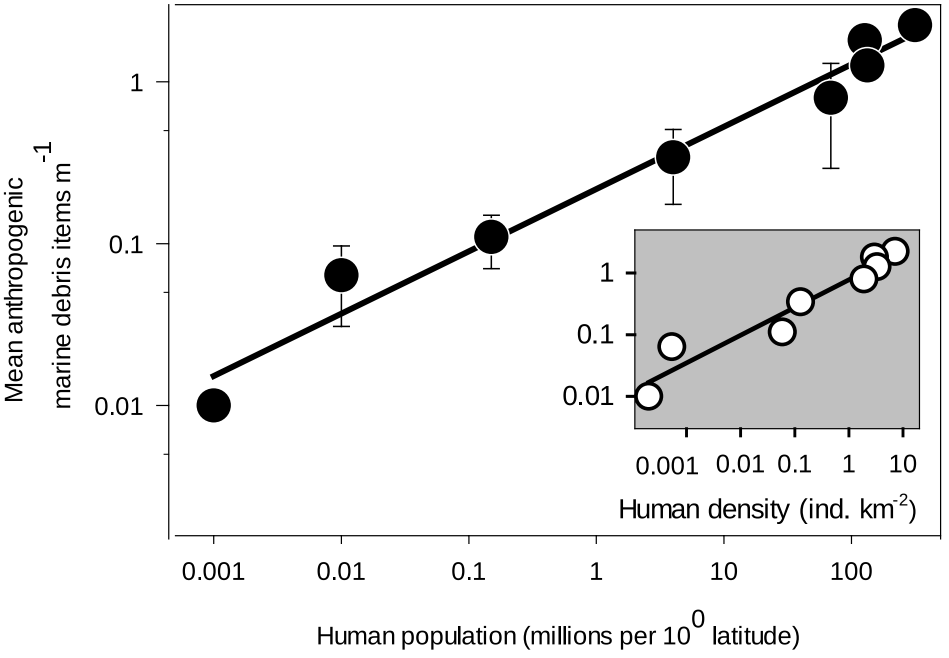

**FIGURE 5.** Mean beach accumulation of debris with human population per 10 degrees latitude in the southern hemisphere. Data presented as mean with standard deviation, calculated from sources as Fig. 1. Main plot is population ($r^2$ = 0.973) and insert is density ($r^2$ = 0.93). Both regressions are significant at $p <$ 0.01 level with ANOVA.

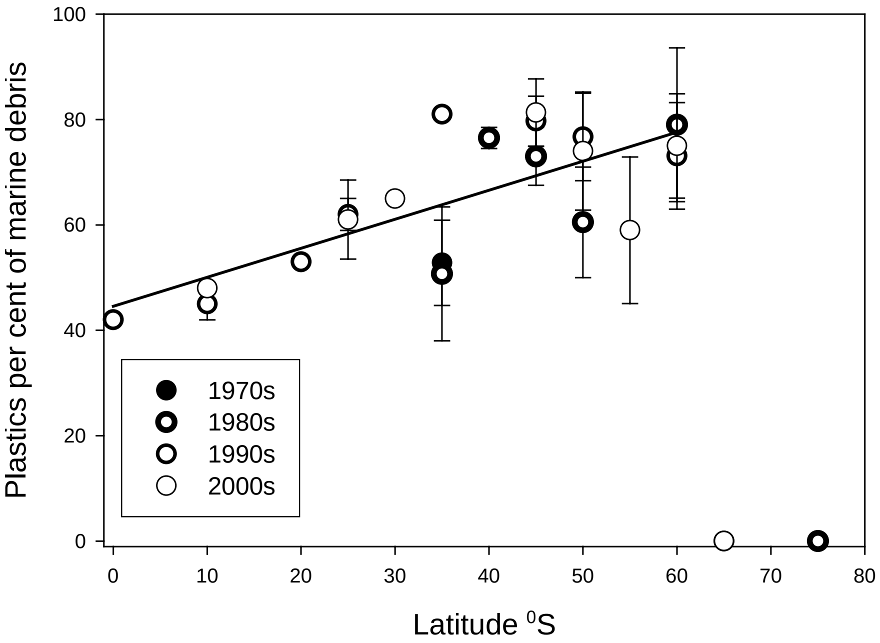

**FIGURE 6**. Proportion (%) of marine debris constituted by plastics with latitude. Data (sources as for Fig. 2) are presented as mean and standard error for each 5 degrees latitude. Line of best fit (regression) has $r^2$ = 55.6 and significance of ANOVA, 1 df, F ratio = 23.8, $p < 0.001$. Data south of 65 degrees not included in analysis.

high proportion of plastics did, especially in the Southern Ocean, actually originate in local or regional fisheries[18,19]. There was no evidence of any change in the proportion of plastics in debris with time (Fig. 6), thus the levels of plastic must, therefore, be increasing at the rate of other marine rubbish (Fig. 4).

The data presented here would indicate that the 1982 estimate of 8 million items estimated to be entering the oceans each day[4] now needs to be multiplied severalfold to be updated. The environmental and economic implications of accelerating debris accumulation and export of plastic flotsam would seem to be considerable. In terms of increased opportunities for invasional pest species alone, the problem of marine artifacts seems daunting. Although both densities (Fig. 2) and organismal colonization rates of debris[8] were lowest in the Southern Ocean, rates of accumulation of debris are increasing fastest (Fig. 4). Furthermore, a recent study[20] found debris floating at 68°S in the Southern Ocean, which was not only colonized by many types of animals, but also evidence that some had survived through an Antarctic winter at the freezing air-sea interface. Anthropogenic debris has been estimated to more than treble chances of faunal propagation in subpolar/polar waters compared to potential travel opportunities on natural debris[8]. Action and implementation of existing dumping legislation (e.g., MARPOL, Annex V) are desperately needed, but we must add concepts such as ‘pristine’, ‘remote’, and ‘wilderness’ to extinction lists. Some surveys have involved the first known visit by man to very ‘remote’ shores, but our miracle material had long since beaten us there.

### REFERENCES

1. Benton, T.G. (1991) Oceans of garbage. *Nature* **352**, 113.
2. Ryan, P.G. and Moloney, C.L. (1993) Marine litter keeps increasing. *Nature* **361**, 23.
3. Gregory, M.R., Kirk, R.M., and Mabin, M.C.G. (1984) Pelagic tar, oil, plastics and other litter in surface waters of the New Zealand sector of the Southern Ocean and on Ross Dependency shores. *N. Z. Antarct. Rec.* **6**, 12–28.

4. Horseman, P.V. (1982) The amount of garbage pollution from merchant ships. *Mar. Poll. Bull*. **13**, 167–169.
5. Thompson, R.C., Olsen, Y., Mitchell, R.P., Davis, A., Rowland, S.J., John, A.W.G., McGonigle, D., and Russel, A.E. (2004) Lost at sea: where is all the plastic? *Science* **304**, 838.
6. Barnes, D.K.A. (2004) Natural and plastic flotsam stranding in the Indian Ocean. In *The Effects of Human Transport on Ecosystems: Cars and Planes, Boats and Trains*. Davenport, D. and Davenport, J.L., Eds. Royal Irish Academy, Dublin. pp. 193–205.
7. Barnes, D.K.A. and Milner, P. (2005) Drifting plastic and its consequences for sessile organism dispersal in the Atlantic Ocean. *Mar. Biol*. **146**, 815–825.
8. Barnes, D.K.A. (2002) Invasions by marine life on plastic debris. *Nature* **416**, 808–809.
9. Masó, M., Garcés, J., Pagès, F., and Camp, J. (2003) Drifting plastic debris as a potential vector for dispersing Harmful Algal Blooms (HAB) species. *Sci. Mar.* **67**, 107–111
10. Barber, H.N., Dadswell, H.E., and Ingle, H.D. (1959) Transport of driftwood from South America to Tasmania and Macquarie Island. *Nature* **184**, 203–204.
11. Coombs, D.S. and Landis, C.A. (1966) Pumice from the South Sandwich eruption of March 1962 reaches New Zealand. *Nature* **209**, 289–290.
12. Gregory, M.R. and Ryan, P.G. (1997) Pelagic plastics and other seaborne persistent synthetic debris. In *Marine Debris*. Coe, J.M. and Rogers, D.B., Eds. Springer. pp. 49–66.
13. Ryan, P.G. and Swanepoel, D. (1996) Cleaning beaches: sweeping the rubbish under the carpet. *S. Afr. J. Sci.* **92**, 275–276.
14. Hayward, B.W. (1984) Rubbish trends – beach litter surveys at Kanerua, 1974–1982. *TANE* **30**, 209–217.
15. Gregory, M.R. (1987) Plastics and other seaborne litter on the shores of New Zealand's Subantarctic islands. *N. Z. Antarct. Rec.***7**, 32–47.
16. Ryan, P.G. (1987) The origin and fate of artefacts stranded at islands in the African sector of the Southern Ocean. *Environ. Cons.* **14**, 341–346.
17. Torres, D. and Jorquera, D. (1999) Synthesis of Marine Debris Survey at Cape Shirreff, Livingston Island, During the Antarctic Season 1998/99. CCAMLR-XVIII/BG/39. CCAMLR, Hobart, Australia.
18. Convey, P., Barnes, D.K.A., and Morton, A. (2002) Debris accumulation on oceanic island shores of the Scotia Arc, Antarctica. *Polar Biol*. **25**, 612–617.
19. Eriksson, C. and Burton, H. (2001) Collections (1991 to 2000) of Marine Debris on Macquarie Island Show Increases in Some Fisheries-Sourced Materials. Abstract S6P07in VIII SCAR International Biology Symposium, Amsterdam, The Netherlands.
20. Barnes, D.K.A. and Fraser, K.P.P. (2003) Rafting by five phyla on man-made flotsam in the Southern Ocean. *Mar. Ecol. Prog. Ser.* **262**, 289–291.

**This article should be referenced as follows:**

Barnes, D.K.A. (2005) Remote islands reveal rapid rise of southern sea debris. *TheScientificWorldJOURNAL* **5**, 915–921. DOI 10.1100/tsw.2005.120.

**Handling Editor:**

R. Kenchington, Associate Editor for *Marine Systems* — a domain of *TheScientificWorldJOURNAL*.
